# Combinatorial Metabolic Engineering and Enzymatic Catalysis Enable Efficient Production of Colanic Acid

**DOI:** 10.3390/microorganisms10050877

**Published:** 2022-04-22

**Authors:** Suwei Li, Xianhao Xu, Xueqin Lv, Yanfeng Liu, Jianghua Li, Guocheng Du, Long Liu

**Affiliations:** 1 Key Laboratory of Carbohydrate Chemistry and Biotechnology, Ministry of Education, Jiangnan University, Wuxi 214122, China; lisuwei@stu.jiangnan.edu.cn (S.L.); xianhaoxu@jiangnan.edu.cn (X.X.); lvxueqin@jiangnan.edu.cn (X.L.); yanfengliu@jiangnan.edu.cn (Y.L.); lijianghua@jiangnan.edu.cn (J.L.); gcdu@jiangnan.edu.cn (G.D.); 2 Science Center for Future Foods, Jiangnan University, Wuxi 214122, China

**Keywords:** colanic acid, RCS system, colanic acid hydrolase

## Abstract

Colanic acid can promote the lifespan of humans by regulating mitochondrial homeostasis, and it has widespread applications in the field of health. However, colanic acid is produced at a low temperature (20 °C) with low titer. Using *Escherichia coli* K-12 MG1655, we constructed the SRP-4 strain with high colanic acid production at 30 °C by enhancing the precursor supply and relieving the regulation of transcription for colanic acid synthesis genes by the RCS system. After media optimization, the colanic acid titer increased by 579.9-fold and reached 12.2 g/L. Subsequently, we successfully purified the colanic acid hydrolase and reduced the molecular weight of colanic acid (106.854 kDa), thereby eliminating the inhibition of high-molecular-weight colanic acid on strain growth. Finally, after adding the colanic acid hydrolase (4000 U/L), the colanic acid with low molecular weight reached 24.99 g/L in 3-L bioreactor, the highest titer reported so far. This high-producing strain of colanic acid will promote the application of low-molecular-weight colanic acid in the field of health.

## 1. Introduction

Colanic acid, an anionic heteropolysaccharide, is secreted by *Enterobacteriaceae* [1,2]. In the 1960s, Goebel first isolated it from the *Escherichia coli* K235 strain and named it colanic acid [3]. The repeating unit of colanic acid is comprised of D-galactose, L-fucose, D-glucose, and D-glucuronic acid, which exist in a molar ratio of 2:2:1:1, and colanic acid is further modified with pyruvate substituents and nonstoichiometric acetyl groups [4,5,6,7]. As a polysaccharide, the molecular weight of colanic acid is variable, and its weight is more than ten million Daltons [8]. Due to its high molecular weight and numerous hydrophilic groups on the surface, colanic acid has excellent water retention; thus, it has widespread applications in cosmetics [8]. In addition to this property, colanic acid has important value in health care products. Two recent articles reported that small-molecular-weight colanic acid fed to *C. elegans* significantly improved lifespan [9]. Further studies have demonstrated that colanic acid can modulate mitochondrial homeostasis and protein processing in host cells, which are conserved across species [9,10]. Therefore, colanic acid has potential applications in the pharmaceutical industry.

**Figure 1:**
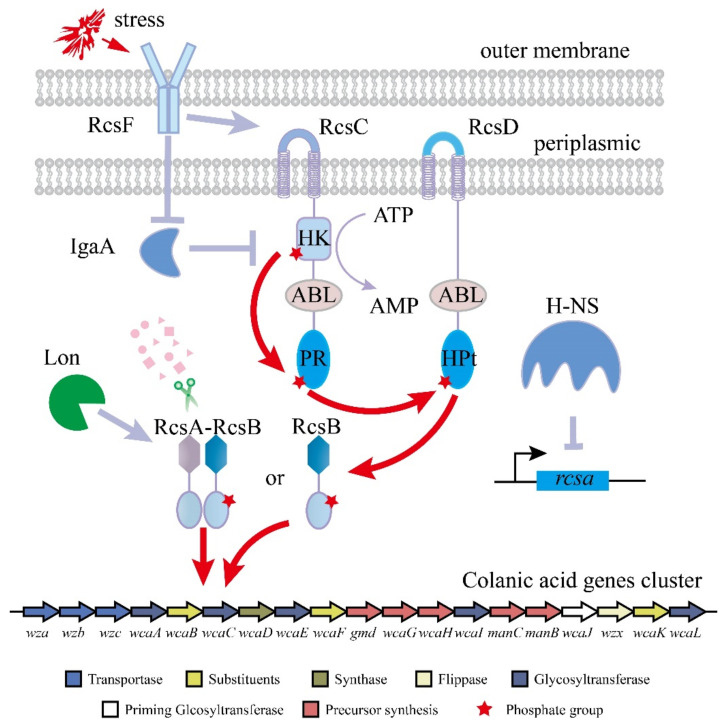
The RCS phosphorylation system. RcsF senses the external stimulus and promotes the phosphorylation of RcsC. Through RcsC and RcsD, the phosphorylation signal is transmitted to RcsB. The phosphorylated RcsB binds to the colanic acid gene cluster alone or after binding to RcsA to promote the transcription of the colanic acid gene cluster.

The production of colanic acid by *E. coli* is induced by environmental stress such as osmotic shock, temperature, oxidative stress, and antibiotics [11,12,13], in which the regulator of capsule synthesis (RCS) phosphorylation system plays important roles (Figure 1) [14,15,16]. In many cases, the researchers turned on the RCS system by culturing the strain at a low temperature, thereby activating the synthesis of colanic acid [14]. This extreme fermentation condition limits the industrial production of colanic acid. The RCS phosphorylation system consists of three core proteins, namely, the response regulator RcsB, the membrane-bound sensor kinase RcsC, and the membrane-bound phosphotransfer protein RcsD [16,17,18]. RcsF, IgaA, and RcsA are also involved in this system [19,20,21]. After exposure to extracellular stress, RcsF is induced, resulting in its interaction with RcsC. Subsequently, RcsC is autophosphorylated at its histidine kinase (HK) domain, and the phosphoryl group is transferred to the phosphoryl receiver (PR) domain, which is an ATP-consuming process [18]. The phosphoryl group is then transferred to the histidine-containing phosphotransmitter (HPt) domain of RcsD and received by RcsB [22]. The phosphorylated RcsB can activate the transcription of *wza* gene cluster, which contains 20 genes (*wza*, *wzb*, *wzc*, *wcaA*, *wcaB*, *wcaC*, *wcaD*, *wcaE*, *wcaF*, *gmd*, *wcaG*, *gmm*, *wcaI*, *manC*, *manB*, *wcaJ*, *wzxC*, *wcaK*, *wcaL,* and *wcaM*) [23,24]. ManB, Manc, Gmd and WcaG are involved in the biosynthesis of GDP-fucose [25]. WcaJ, WcaI, WcaF, WcaE, WcaC, WcaB, WcaA, WcaL, and WcaK are involved in the biosynthesis of the repeating unit of colanic acid [26,27]. Wza, Wzb, Wzc, WzxC, and Wcad are involved in the polymerization and export of colanic acid [13,28,29].

Furthermore, IgaA can negatively regulate RcsC activity, but the interaction between these proteins is still unclear [20]. RcsA, an unstable sensor kinase, cooperates with RcsB to regulate the transcription of the *wza* gene cluster, but its level is usually low because RcsA is degraded by Lon protease [30], and the *rcsa* gene is suppressed by H-NS [31]. Therefore, it is necessary to relieve the regulation of transcription for colanic acid synthesis genes by the RCS system to increase the fermentation temperature and the production of colanic acid.

Research related to colanic acid now focuses on the link between lipopolysaccharide and colanic acid [32,33]. Ren et al. constructed several mutant strains with different lipopolysaccharide structures by knocking out the genes related to lipopolysaccharide synthesis in *E. coli* and verifying the effects of different lipopolysaccharide structures on the production of colanic acid; however, the titer of colanic acid was still very low (0.27 mg/L) [32]. Through fermentation optimization, Han et al. increased the colanic acid titer of the *E. coli* K-12 Δ*waaf* strain to 2.05 g/L [34]. In 2019, Wu et al. further increased the titer of colanic acid to 10.39 g/L by expressing polyhydroxybutyrate (PHB) synthase [8]. However, the molecular weight of colanic acid reported in the above articles was greater than 10 MDa, indicating it has no physiological activity [9]. The high concentration of colanic acid also increases the viscosity of the solution, which prevents further improvement of the colanic acid titer. Therefore, it is crucial to reduce the molecular weight of colanic acid to enhance its production.

In this work, to achieve the high titer of low molecular weight colanic acid, we firstly increased the fermentation temperature of colanic acid from 20 °C to 30 °C by relieving the regulation of RCS phosphorylation system on colanic acid synthesis, thereby reducing the production cost of colanic acid. Subsequently, the synthesis efficiency of colanic acid was improved by media optimization and enhancing the precursor supply. Finally, by expressing and adding colanic acid hydrolase, the molecular weight of colanic acid was significantly reduced, and low molecular weight colanic acid was obtained. This study provides theoretical guidance for the optimization of the metabolic network of the other polysaccharides.

## 2. Materials and Methods

### 2.1. Bacterial Strains and Plasmids

Plasmids and strains used in this study are listed in Table S1. *E. coli* JM109 was used for plasmid amplification and recombinant plasmid construction. *E. coli* K-12 MG1655 was used as the parental strain. Recombinant strains were all derived from *E. coli* K-12 MG1655. The colanic acid hydrolase gene (GenBank Accession Number HM214492) was synthesized by GENEWIZ (Suzhou, China), and *E. coli* BL21 (DE3) was used for the expression of colanic acid hydrolase. All primers used in this study are listed in Table S2.

To construct the PACY-CAE plasmid, the PACYDUET1 plasmid was linearized using primers Pacy-T and Pacy-B, and the synthesized colanic acid hydrolase gene was amplified using primers CAE-T and CAE-B. These two DNA fragments were ligated using the Gibson Assembly Cloning Kit (NEB) to generate the PACY-CAE plasmid. Finally, it was transformed into *E. coli* BL21(DE3), generating the BL21-CAE strain.

The Clustered Regularly Interspaced Short Palindromic Repeats/Cas9 (CRISPR/Cas9) system was employed to delete and overexpress the genes in the chromosomes of *E. coli* strains. For example, to construct the SP-1 strain, upstream and downstream sequences of the *flie* gene were amplified using the primer pair T-*flie*-L/T-*flie*-R and D-*flie*-L/D-*flie*-R, respectively. The *galu* gene was amplified using the primer pair M-*galu*-L/M-*galu*-R. An overlapping PCR was then employed to link these fragments using primers T-*flie*-L and D-*flie*-R. The pTarget-*flie* plasmid, which was derived from pTarget, was constructed using the primer pair pTarget-*flie*-L/pTarget-*flie*-R. Finally, the fused DNA segment and the pTarget-*flie* plasmid were co-transferred into *E. coli* K-12/pCas9 competent cells by electroporation (2000 V, 200 Ω, 25 µF, 4 ms pulse duration). The recombinant strains were confirmed by colony PCR and DNA sequencing. The pTarget-*flie* plasmid and pCas9 in the recombinant strain were isolated by adding isopropyl-β-D-thiogalactoside (IPTG) and culturing cells at 42 °C overnight, respectively. The other recombinant strains were constructed using the aforementioned method and the corresponding primer pairs.

### 2.2. Medium and Cultivation

The recombinant strains for colanic acid production were firstly cultivated in 3 mL LB medium (5 g/L yeast extract, 10 g/L tryptone, and 10 g/L NaCl) in a 14 mL tube set at 30 °C and 220 rpm. Then, 0.3 mL seed solution was inoculated in 30 mL M9 medium (4 g/L glucose, 17.1 g/L Na_2_HPO_4_·12H_2_O, 3 g/L KH_2_PO_4_, 0.5 g/L NaCl, 1 g/L NH_4_Cl, 0.24 g/L MgSO_4_, and 0.011 g/L CaCl_2_), LB medium, SOB medium (20 g/L tryptone, 5 g/L yeast extract, 0.5 g/L NaCl, 0.186 g/L KCl, and 0.95 g/L MgCl_2_), TB medium (24 g/L yeast extract, 12 g/L tryptone, 4 mL/L glycerin, 2.31 g/L KH_2_PO_4_, and 12.54 g/L K_2_HPO_4_), or TBG medium (24 g/L yeast extract, 12 g/L tryptone, 4 mL/L glycerin, 2.31 g/L KH_2_PO_4_, 12.54 g/L K_2_HPO_4_, and 20 g/L glucose) in 250 mL shake flasks and cultivated at 30 °C and 220 rpm for 48 h. Different amounts of colanic acid hydrolase (CAE) and appropriate antibiotics (50 mg/L kanamycin, 30 mg/L chloramphenicol, and 100 mg/L spectinomycin) were added to the culture medium when needed.

The fed-batch cultivation of *E. coli* was performed in a 3-L bioreactor with 1.5 L of TB medium. In brief, the seed culture was prepared in 150 mL TB medium in 500 mL shake flasks set at 30 °C and 220 rpm for 12 h. Then, the 150 mL seed culture was inoculated in the 3-L bioreactor with 1.5 L of TB medium. At 9 h, 10 g/L glucose was supplemented and maintained in the range of 8–13 g/L. The glucose concentration was measured by M-100 Biosensor Analyzer (SIEMAN, Shenzhen, China) at 12 h, 20 h, 28 h, 36 h, 44 h, 52 h, 60 h, and 72 h. The dissolved oxygen level of the bioreactor was maintained at 30%. An ammonia solution (50%, *v*/*v*) was automatically fed to maintain the culture pH in the range of 6.8–7.0, and 4000 U/L colanic acid hydrolase was added to the culture medium when colanic acid began to accumulate at the exponential phase.

### 2.3. Expression and Enzyme Activity of Colanic Acid Hydrolase

To express colanic acid hydrolase, the BL21-CAE strain was grown in 100 mL LB medium containing 30 mg/L chloramphenicol in a 500 mL shake flask set at 37 °C and 220 rpm. When cultures reached the OD_600_ of 0.5, 0.015 mM IPTG was added, and the temperature was changed to 16 °C. After 20 h, the growth medium was centrifuged at 5000× *g* for 5 min, and the cells were collected. After ultrasonication and centrifugation, the supernatant was collected and purified on a Ni-NTA column. In brief, the supernatant was loaded onto an equilibrated His GraviTrap column (GE Healthcare, Boston, MA, USA) and purified by ÄKTA FPLC (GE Healthcare). Subsequently, the column was washed with buffer A (500 mM NaCl, 20 mM PB, 20 mM imidazole, pH 8.0) until the OD_280_ was stable. Colanic acid hydrolase was eluted with 20% buffer B (500 mM NaCl, 20 mM PB, 500 mM imidazole, pH 8.0). Finally, the eluted colanic acid hydrolase was dialyzed against 20 mM PB (pH 8.0) to remove imidazole, and the purified colanic acid hydrolase was stored at 4 °C for further use.

The DNS method was used to measure enzyme activity [35]. In brief, 500 μL of colanic acid (10 g/L) was mixed with 500 μL of colanic acid hydrolase solution. Then, the sample was hydrolyzed at 37 °C for 30 min. After adding 750 μL of DNS reagent (6.3 g/L 3,5-dinitrosalicylic acid, 20.96 g/L NaOH, 182 g/L potassium sodium tartrate, 5 g/L phenol, 5 g/L Na_2_SO_3_), the solution was boiled for 5 min. After cooling, 1.75 mL of deionized water was added to the sample. The absorbance was measured at 540 nm. Colanic acid (10 g/L) was used as the control, and different concentrations of glucose were used to generate the standard curve.

### 2.4. Purification and Acid Hydrolysis of Colanic Acid

To obtain purified colanic acid, the recombinant strains were grown in 100 mL M9 medium in a 500 mL shaking incubator set at 30 °C for 48 h. After boiling the cultures for 20 min, the cells were removed by centrifugation, and the supernatant was collected. A total of 300 mL ethanol was added into the supernatant, and the sample was stored at 4 °C for overnight. The sample was centrifuged, and the precipitate was collected. After dissolving the pellets in 100 mL ultra-pure water, 1 mL acetic acid was added into the solution, which was then boiled for 2 h and centrifuged at 10,000× *g* at 4 °C for 30 min to precipitate the lipopolysaccharides. Then, the supernatant was mixed with 100 mL trichloromethane/n-butanol (4:1 *v*/*v*) to remove proteins. The water phase was collected and dialyzed against water using a 14,000-Da dialysis cassette for 2 d. Finally, purified colanic acid was obtained after lyophilization. The average molecular weight of colanic acid was determined by Waters 1525 size-exclusion chromatography with a 2410 refractive index detector (GFPC) after injecting 20 µL of colanic acid (5 g/L). The flow rate was 0.9 mL/min, and the mobile phase was 0.1 mol/L NaAc.

To determine the monosaccharide composition of the polysaccharides, 2 mol/L trifluoroacetate (TFA) was used to hydrolyze colanic acid. In brief, 3.5 mg of purified colanic acid was added to 250 µL of 2 mol/L TFA and boiled at 100 °C for 2 h. After drying with nitrogen and dissolving in 250 µL double-distilled H_2_O, the sample was analyzed by thin-layer chromatography (TLC) and high-performance liquid chromatography (HPLC). High-performance ion-exchange chromatography (Dionex ICS-5000, Waltham, MA, USA) was performed with a Carbo PA20 column (3 mm × 150 mm). From 0 to 21 min, the mobile phase was 97.4% water and 2.6% 250 mM NaOH. From 21 to 30 min, the mobile phase was 77.4% water, 2.6% 250 mM NaOH, and 20% 1 M NaAc. From 30 to 50 min, the mobile phase was 20% water and 80% 250 mM NaOH. The flow rate was 0.5 mL/min. The standard sample was comprised of L-fucose, D-galactose, D-glucose, and D-glucuronic acid. TLC was performed with a silica TLC plate (gel 60 F254, Merck, Darmstadt, Germany) using n-butanol/ethanol/water (2:2:1 *v*/*v*/*v*) as the developing solvent and lichenol reagent as the chromogenic agent.

### 2.5. Quantification of Colanic Acid

The fermentation broth was boiled for 20 min to dissolve the colanic acid. The solution was centrifuged at 10,000× *g* for 30 min. The supernatant was collected; 0.5 mL supernatant was mixed with 0.5 mL 4 mol/L TFA. The sample was hydrolyzed at 100 °C for 4 h. Subsequently, the hydrolysate was evaporated in a vacuum and reconstituted with 0.5 mL water. The fucose content was measured by HPLC using an Aminex HPX-87H column, and 5 mM H_2_SO_4_ was used to separate the sample at a rate of 0.6 mL/min at 55 °C. The fermentation broth of the strain with the deleted *wcaj* gene was used as the control. A standard curve was generated using different concentrations of fucose, and the content of colanic acid was calculated from the different concentrations of fucose.

### 2.6. RNA Extraction and Real-Time PCR

All qPCR primers used in this study are listed in Table S2. Total RNA was extracted from *E. coli* using the RNA Simple Total RNA Kit (Tiangen, Beijing, China) and reverse transcribed into cDNA using the TianSeq M-MLV (RNase H) Kit (Tiangen). The mRNA expression levels of the target genes were measured by the FastFire qPCR PreMix (SYBR Green) Kit (Tiangen) using the StepOnePlus System (Applied Biosystems, Waltham, MA, USA). The expression levels were analyzed using the 2^−^^ΔΔCT^ method, and the *reca* gene was used as a control [36]. Three parallel samples were assayed.

### 2.7. Enzymatic Hydrolysate Detection

The purified colanic acid was dissolved in double-distilled H_2_O. Subsequently, 10 mL of colanic acid (10 g/L) was added to 10 mL of enzyme solution (120,000 U/L) and hydrolyzed at 37 °C for 24 h to generate the CAhy-1 sample. Then, 20 mL trichloromethane/n-butanol (4:1 *v*/*v*) was added into the CAhy-1 sample to remove proteins. After centrifugation, the water phase was collected and dialyzed against water using a 500-Da dialysis cassette for 2 d. The colanic acid hydrolysate was obtained by lyophilization and redissolved in double-distilled H_2_O to generate the CAhy-2 sample. The CAhy-1 sample was assayed by TLC and HPLC, whereas the CAhy-2 sample was assayed by matrix-assisted laser desorption/ionization time-of-flight mass spectrometry (MALDI-TOF-MS) (Ultrafle Xtreme, Bruker Daltonics, Billerica, MA, USA).

### 2.8. Atomic Force Microscopy

The morphology of purified colanic acid was observed by atomic force microscopy (AFM). Briefly, 10 µL of colanic acid (5 g/L) was placed on a mica sheet and observed after drying at room temperature. AFM images were analyzed with Nanoscope 1.9 Software.

### 2.9. Statistical Analysis

Three independent replicates were performed for all experiments. Statistical data analysis was performed with t-texts in SPSS 25.0. *p* values of <0.05 were considered statistically significant, and statistical significance is indicated as * for *p* < 0.05 and ** for *p* < 0.01.

## 3. Results

### 3.1. Characterization of Colanic Acid

**Figure 2:**
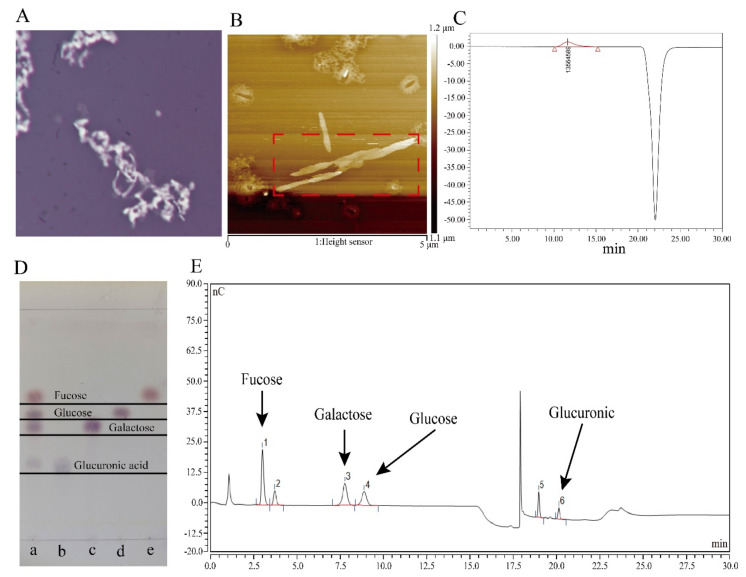
Characterization of colanic acid. (**A**) Image of *Escherichia coli* K-12 cells with colanic acid capsule observed by microscope. (**B**) Atomic force microscopy results of the colanic acid polysac-charide. In the red box was colanic acid polysaccharide. (**C**) Evaluation of the molecular weight of colanic acid by high-performance gel liquid chromatography. (**D**) Thin layer chromatography re-sults of the colanic acid hydrolyzate. Lane a was the hydrolyzed sample. Lanes b, c, d, and e were glucuronic acid, galactose, glucose, and fucose standards, respectively. (**E**) Ionic liquid chroma-tography results of the colanic acid hydrolysate. Peaks 1, 3, 4 and 6 were fucose, galactose, glucose and glucuronic acid, respectively.

For colanic acid production, *E. coli* is usually grown in M9 medium at low temperature (20 °C). When wild-type *E. coli* K-12 was cultured on M9 medium plates at 20 °C, we found that its colony morphology changed. After staining with nigrosine and observing by microscopy, the edges of the cells were irregular in shape (Figure 2A). Therefore, to explore whether the substance around the cells was colanic acid, we fermented the wild-type *E. coli* K-12 cell; after the extraction and purification, we obtained the corresponding substance.

After observing by atomic force microscopy (AFM) (Figure 2B), the substance had a sharp-like property with multiple helices, which is consistent with the properties of polysaccharides [37]. The molecular weight of the substance was greater than 10 MDa as measured by high-performance gel liquid chromatography (GFPC) (Figure 2C), consistent with a previous study that reported the molecular weight of colanic acid [8]. Subsequently, to further confirm that the polysaccharide was colanic acid, we used trifluoroacetate (TFA) to hydrolyze the polysaccharide, followed by thin-layer chromatography (TLC) (Figure 2D) and ionic liquid chromatography (HPLC) (Figure 2E) to determine the monosaccharide composition of the polysaccharide and the corresponding ratio of monosaccharide moles, respectively. The results showed that the polysaccharide was comprised of L-fucose, D-glucose, D-galactose, and D-glucuronic acid, which is consistent with the monosaccharide composition of colanic acid. The molar ratio of L-fucose, D-glucose, D-galactose, and D-glucuronic acid was calculated as 13.4:6.2:11.4:4.3, which is close to the theoretical value of colanic acid [3,4,5].

The *wcaj* gene is the initiator of colanic acid synthesis [27]. So, we deleted the *wcaj* (undecaprenyl-phosphate glucose-1-phosphate transferase) gene in the *E. coli* K-12 strain to construct the SC-1 strain. After deleting the gene, we measured the colanic acid titers of *E. coli* K-12 and SC-1 strain according to the measurement method of colanic acid, and the *E. coli* K-12 produced 20 mg/L of colanic acid, whereas the SC-1 did not produce colanic acid. Taken collectively, these results indicate that the substance was colanic acid.

### 3.2. Relieving the Regulation of the RCS Phosphorylation System on the Synthesis of Colanic Acid and Optimizing the Fermentation Medium

**Figure 3:**
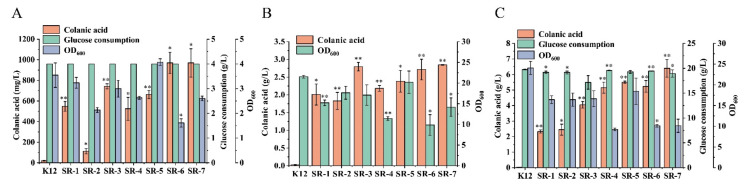
Relieving the regulation of the RCS phosphorylation system on the synthesis of colanic acid and the optimization of medium. (**A**) Colanic acid titers of SR recombinant strains in M9 medium at 30 °C in shake flasks. (**B**) Colanic acid titers of SR recombinant strains in TB medium at 30 °C in shake flasks. (**C**) Colanic acid titers of SR recombinant strains in TBG medium at 30 °C in shake flasks. Statistical data analysis was performed with t-texts in SPSS 25.0. *p* values of <0.05 were considered statistically significant, and statistical significance is indicated as * for *p* < 0.05 and ** for *p* < 0.01.

In the *E. coli* K-12 strain, genes related to colanic acid synthesis are located in a gene cluster, and the gene cluster is regulated by the RCS phosphorylation system (Figure 1). When the strain is in an unfavorable growth environment, such as low temperature, the RCS phosphorylation system will respond to external stimuli and turn on the transcription of the colanic acid gene cluster. However, low temperature affects strain growth, which seriously affects colanic acid production. To construct a chassis cell with high colanic acid production, we relieved the regulation of the RCS phosphorylation system on the colanic acid gene cluster. We constructed SR-1, SR-2, and SR-3 strains by deleting genes *lon* and *hns* and using P*_tac_* promoter to overexpress the *rcsa* gene in the wild-type *E. coli* K-12 strain, respectively. Compared with the wild-type strain, we found that the constructed strains can be fermented in M9 medium at 30 °C to produce colanic acid. After cultured in 30 mL M9 medium in shake flasks at 30 °C for 48 h, the colanic acid titers of SR-1, SR-2, and SR-3 strains were 548 mg/L, 113 mg/L, and 742 mg/L, respectively (Figure 3A). Subsequently, we combined these genes to construct the following strains: SR-4 (Δ*lon* Δ*hns*), SR-5 (Δ*lon mota*::*rcsa*), SR-6 (Δ*hns mota*::*rcsa*), and SR-7 (Δ*lon* Δ*hns mota*::*rcsa*). The colanic acid titers of SR-4 and SR-5 strains were 524.5 mg/L and 663.5 mg/L, respectively, whereas the titers of SR-6 and SR-7 strains were identical at 970 mg/L (Figure 3A). We used a real-time quantitative PCR to detect the mRNA level of the *wza* gene, which is the first gene in the colanic acid gene cluster. The relative mRNA level of the *wza* gene in the SR-7 strain increased 2.61-fold compared with the original *E. coli* K12 strain (Table 1). These results confirmed that we successfully relieved the regulation of RCS phosphorylation system on the transcription of colanic acid gene cluster.

In M9 medium, colanic acid production by SR-1 to SR-7 strains was higher than that of the wild-type strain, but the OD_600_ values of these strains were lower. The OD_600_ values of strains SR-1 to SR-7 were 3.23, 2.13, 2.99, 2.62, 4.07, 1.61, and 2.60, respectively (Figure 3A), indicating they are not suitable for high colanic acid production. Therefore, we tried several medium, including LB, SOB, and TB, to improve cell growth. Attempts in both LB (Supplementary Figure S1A) and SOB (Supplementary Figure S1B) media were unsuccessful, with no positive results for the strain growth and the production of colanic acid. Attempts in TB medium were successful. In TB medium, the growth of the strains was improved, and the OD_600_ values of SR-1 to SR-7 were 15.2, 17.6, 17.1, 11.4, 20.2, 9.8, and 14.1, respectively (Figure 3B). Compared with M9 medium, the colanic acid titers of SR-1 to SR-7, strains increased by 2.66-fold, 15-fold, 2.76-fold, 3.16-fold, 2.59-fold, 1.79-fold, and 1.93-fold and reached 2.01 g/L, 1.82 g/L, 2.79 g/L, 2.18 g/L, 2.38 g/L, 2.71 g/L, and 2.84 g/L, respectively (Figure 3B). We also found that the wild-type *E. coli* K-12 strain produced colanic acid when fermented in TB medium at 30 °C due to the improved growth conditions, but the titer was low (25 mg/L) compared with the recombinant strains (Figure 3B). To further increase the titer of colanic acid, we supplemented the TB medium with glucose (20 g/L), which served as a carbon source, generating TBG medium. After fermentation, except for SR-1 and SR-2 strains, the colanic acid titers of the other strains increased significantly. Compared with TB medium, the colanic acid titers of SR-3 to SR-7 strains increased by 0.44-fold, 1.35-fold, 1.31-fold, 0.92-fold, and 1.24-fold in TBG medium, and the colanic titers of SR-1 to SR-7 strains reached 4.05 g/L, 5.15 g/L, 5.51 g/L, 5.23 g/L, and 6.4 g/L, respectively (Figure 3C). The growths of SR-1 to SR-7 strains were affected by glucose (Figure 3C). Therefore, TBG medium is a suitable medium for colanic acid production.

### 3.3. Enhancement of Colanic Acid Production by Overexpression of Precursors

**Figure 4:**
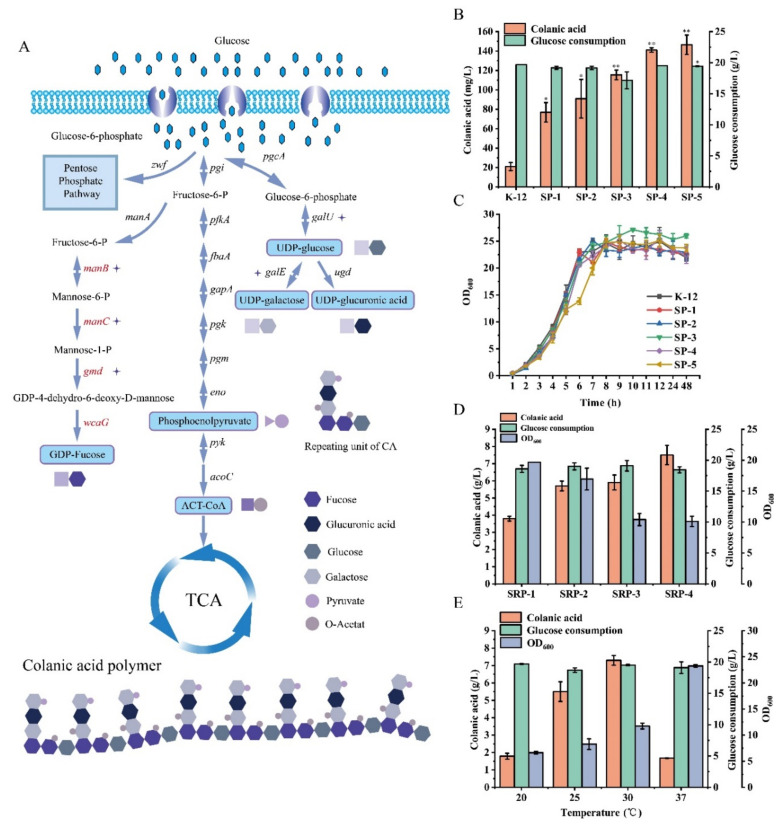
Enhancement of colanic acid production by overexpression of precursors. (**A**) The metabolic pathway of glucose in *Escherichia coli* K-12 and the structure of colanic acid. (**B**) Colanic acid titers of SP recombinant strains in TBG medium at 30 °C in shake flasks. (**C**) Growth curves of SP recombinant strains in TBG medium at 30 °C in shake flasks. (**D**) Colanic acid titers of SRP recombinant strains in TBG medium at 30 °C in shake flasks. (**E**) Colanic acid titers of the SRP-4 strain in TBG medium at different temperatures in shake flasks. Statistical data analysis was performed with t-texts in SPSS 25.0. *p* values of <0.05 were considered statistically significant, and statistical significance is indicated as * for *p* < 0.05 and ** for *p* < 0.01.

To explore the effects of colanic acid precursors on the synthesis of colanic acid and to increase the titer of colanic acid, we overexpressed the genes related to the synthesis of colanic acid precursors. Colanic acid is composed of six precursors, namely, UDP-glucose, UDP-galactose, UDP-glucuronic acid, GDP-fucose, acetyl-CoA, and phosphoenolpyruvate (Figure 4A). Of these, UDP-glucose, UDP-galactose, and UDP-glucuronic acid are located in the same metabolic branch. Therefore, we constructed the SP-1 strain by overexpressing the *galu* gene, which upregulate the conversion of glucose 6-phosphate to UDP-glucose, in the *E. coli* K-12 strain. After fermentation in the TBG medium, the production of colanic acid by the SP-1 strain reached 76.9 mg/L, which is 3.66-fold higher than that of the wild-type strain (Figure 4B). We constructed the SP-2 strain by overexpressing the *gale* gene in the SP-1 strain to upregulate the supply of UDP-galactose. After fermentation, the production of colanic acid by the SP-2 strain reached 90.8 mg/L, which was 1.18-fold higher than that of the SP-1 strain (Figure 4B). To upregulate the supply of GDP-fucose, we overexpressed *gmd*, *manc*, and *manb* genes in the SP-2 strain to construct SP-3, SP-4, and SP-5 strains. Compared with the SP-2 strain, the colanic acid titers of SP-3, SP-4, and SP-5 strains increased by 0.26-fold, 0.55-fold, and 0.60-fold, and reached 115 mg/L, 141 mg/L, and 146 mg/L, respectively (Figure 4B). However, the growth of SP-1 to SP-5 strains was not affected (Figure 4C). Although the colanic acid titer of the SP-5 strain was 6.95-fold higher than that of the *E. coli* K-12 strain, it was still low compared with the SR strains. To further improve the colanic acid titer, we relieved the regulation of RCS phosphorylation system on colanic acid production in the SP-5 strain.

We constructed four mutants. SRP-1 was derived from the SP-5 strain by overexpressing the *rcsa* gene. SRP-2 was derived from the SRP-1 strain by deleting the *lon* gene. SRP-3 was derived from the SRP-1 strain by deleting the *hns* gene. SRP-4 was derived from the SRP-2 strain by deleting the *hns* gene. Except for the SRP-1 strain, all strains had higher colanic acid titers than the corresponding SR strains. The colanic acid titers of SRP-2, SRP-3, and SRP-4 strains were 5.7 g/L, 5.9 g/L, and 7.5 g/L, respectively (Figure 4D). The production of colanic acid by the SRP-4 strain was 1.17-fold higher than that of the SR-7 strain, which had the highest colanic acid titer among all the recombinant strains. The growths of SRP-1 to SRP-4 strains were affected (Figure 4D).

Subsequently, we determined the colanic acid titer of the SRP-4 strain at different temperatures. The SRP-4 strain was cultivated at 20 °C, 25 °C, 30 °C, and 37 °C. We found that the optimum fermentation temperature of the SRP-4 strain was 30 °C, and the colanic acid titer was 7.5 g/L, which was significantly higher than that at 20 °C (1.78 g/L) (Figure 4E). Culturing at 20 °C can activate the RCS phosphorylation system with higher efficiency, thereby promoting the synthesis of colanic acid. However, at 20 °C, the physiological activity of the SRP-4 was inhibited. Therefore, the titer of colanic acid was lower than that at 30 °C.

Then, we determined the molecular weight of colanic acid produced by the SRP-4 strain at 30 °C, and it was 8.71 MDa (Table 2), indicating that it had no physiological activity. Due to the high titer of colanic acid with high molecular weight, the fermentation broth of the SRP-4 strain was more viscous than that of the original strain (Supplementary Video S1), making it impossible to further increase the colanic acid titer. Therefore, we needed to identify a way to reduce the molecular weight of colanic acid.

### 3.4. Expression and Characterization of Enzymatic Hydrolysates

As a high-molecular-weight polysaccharide, colanic acid has no physiological activity. To obtain small-molecular-weight colanic acid with physiological activity, we expressed and purified the colanic acid hydrolase derived from the NST1 bacteriophage [38]. To express the colanic acid hydrolase (CAE), we cloned the hydrolase gene into a plasmid under the regulation of IPTG-inducible T7 promoter. The hydrolase has a 6 His-tag at the N-terminus. Then the plasmid was transformed into BL21(DE3), generating the strain BL21-CAE. After IPTG-induced expression, cell disruption and nickel column purification, we successfully obtained the hydrolase with a molecular weight of 73.3 kDa (Supplementary Figure S2).

**Figure 5:**
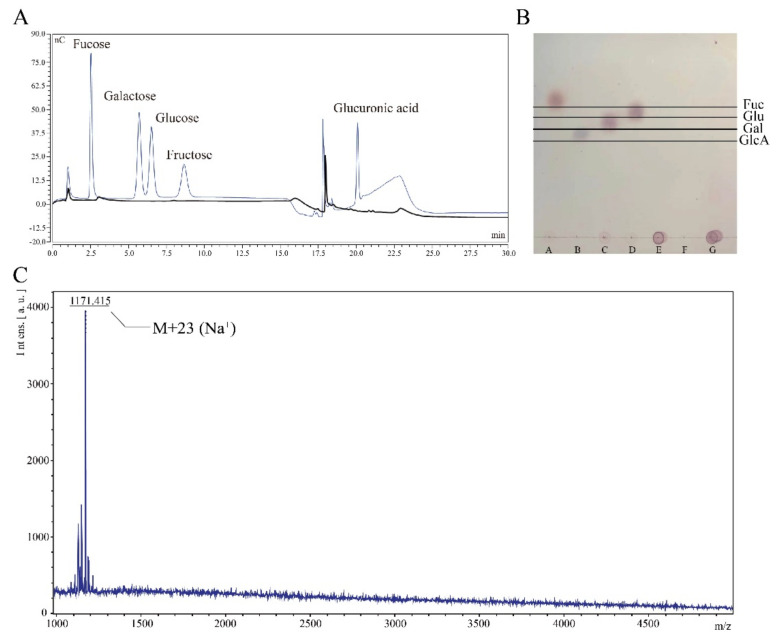
Characterization of enzymatic hydrolysates. (**A**) Purified colanic acid was hydrolyzed by colanic acid hydrolase, and the hydrolysate was assayed by high-performance liquid chromatog-raphy (HPLC). The bold curve was the sample, and the other curve was the standard curve for the different types of monosaccharide. (**B**) Purified colanic acid was hydrolyzed by colanic acid hydro-lase, and the hydrolysate was assayed by thin-layer chromatography (TLC). A, B, C, D, E, F, and G are fucose, glucuronic acid, galactose, glucose, colanic acid, purified colanic acid hydrolase (CAE), and the hydrolysate, respectively. (**C**) The molecular weight of the hydrolysate was determined by matrix assisted laser desorption ion flight mass spectrometry (MALDI-TOF).

Given that the function of this hydrolase was unclear, we analyzed the hydrolyzed product and found that the product was comprised of a repeating polymer unit of colanic acid. Firstly, we added 10 mL enzyme solution (120,000 U/L) into the 10 mL colanic acid solution (10 g/L) to facilitate hydrolysis for 48 h; the solution was named as sample CAhy-1. We assayed sample CAhy-1 by ionic liquid chromatography and did not detect any monosaccharides (Figure 5A). Then, we tested sample CAhy-1 by thin layer chromatography, which did not reveal any monosaccharides and oligosaccharides (Figure 5B). Secondly, we used chloroform/n-butanol (4:1 *v*/*v*) to remove proteins and dialyzed the solution by using a 500-Dalton dialysis bag for 48 h. After freeze-drying, we obtained low-molecular-weight colanic acid. Then, 5 mg low-molecular-weight colanic acid was dissolved in 1 mL double-distilled H_2_O; the solution was named as sample CAhy-2. We used MALDI-TOF to detect the sample, and the molecular weight of the substance was 1,148 Daltons, which was consistent with the molecular weight of the colanic acid repeating unit (Figure 5C). Thus, the hydrolyzate was a repeating polymerized unit of colanic acid.

The hydrolysis of the polysaccharide results in the cleavage of glycosidic bonds, thereby releasing the reduced end. Therefore, we used the 3,5-dinitrosalicylic acid method (DNS) to measure the reduced sugar content. To remove interfering factors, we also measured the reduced sugar content of the colanic acid solution and the enzyme solution, and no reduced sugar was detected. As such, we characterized the enzymatic activity of colanic acid hydrolase. The enzymatic activity to produce 1 ng of reduced sugar (calculated as glucose) in 30 min was 1 U.

### 3.5. Colanic Acid Capsule Layer Inhibits Strain Growth and Glucose Acquisition

**Figure 6:**
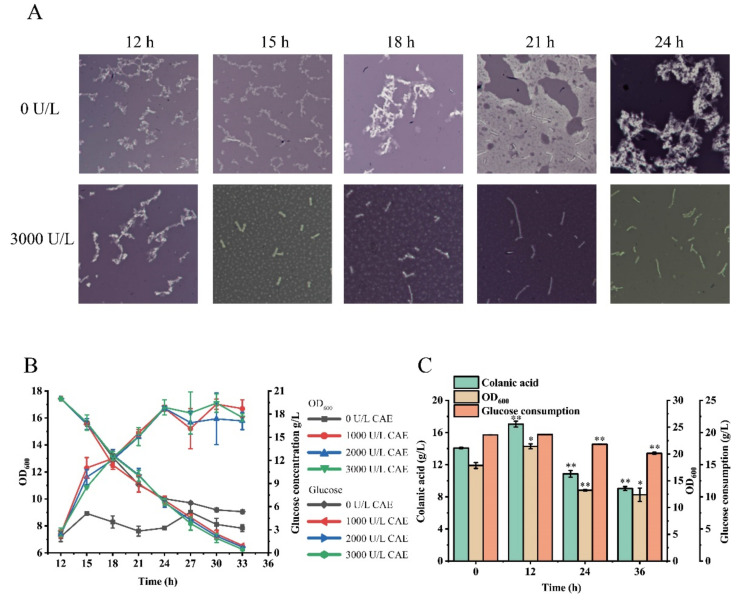
Colanic acid capsule layer inhibited strain growth and glucose acquisition. (**A**) The formation of the colanic acid capsule and the disruption of the colanic acid capsule after adding the hydrolase. (**B**) The growth and glucose consumption of the SRP-4 strain at 30 °C in shake flasks after adding different amounts of colanic acid hydrolase. (**C**) The colanic acid titers of the SRP-4 strain after adding 4000 U/L hydrolase at different times. Statistical data analysis was performed with t-texts in SPSS 25.0. *p* values of <0.05 were considered statistically significant, and statistical significance is indicated as * for *p* < 0.05 and ** for *p* < 0.01.

The fermentation broth of the SRP-4 strain was viscous due to the high colanic acid titer. We examined the morphology of the SRP-4 strain by microscopy and found that a large amount of colanic acid surrounded the individual cell, thereby forming a colanic acid capsule layer (Figure 6A). Several cells were encapsulated in this layer. To explore the effects of the colanic acid capsule layer on strain growth and glucose acquisition, we carried out the following experiment. After 12 h of fermentation in TB medium, 20 g/L glucose was added to the fermentation broth of the SRP-4 strain, followed by 1000 U/L, 2000 U/L (Supplementary Figure S3), and 3000 U/L colanic acid hydrolase, respectively. The OD_600_ and glucose concentration of the strain were measured for every 3 h. After the addition of the hydrolase, we found that the colanic acid capsule layer was disrupted, and the growth of the strain was improved. The OD_600_ value of the sample supplemented with 3000 U/L hydrolase was 16.03, whereas the OD_600_ value of the control sample without hydrolase was approximately 7.83. Therefore, the growth of the control sample was severely inhibited (Figure 6B). In addition, we found that the colanic acid capsule also inhibited glucose acquisition. The glucose consumption rate of the control sample decreased at 24 h. After 33 h of fermentation, 4.6 g/L glucose remained. In the sample with hydrolase, the glucose was completely consumed by 33 h (Figure 6B). By microscopy, the colanic acid capsule layer with hydrolase was disrupted, and white spherical substances were observed uniformly distributed in the field of view. With time, the spherical mass gradually became smaller and eventually became unobservable. However, the colanic acid capsule layer of the control strain was always observed (Figure 6A).

Subsequently, we explored the effect of adding different amounts of colanic acid hydrolase on colanic acid production; we found adding 4000 U/L colanic acid hydrolase provided the best result (Supplementary Figure S4). Then, we added 4000 U/L of hydrolase to the fermentation broth at different times and measured the colanic acid titer and OD_600_ of the strains. The sample with hydrolase added at 12 h had the highest colanic acid production (17.08 g/L). It was 2.27-fold of the control sample (7.5 g/L) without colanic acid hydrolase. The OD_600_ of the sample with hydrolase added at 12 h was also the highest, which was 21.4 (Figure 6C). The samples with hydrolase added at 24 h and 36 h both had less pronounced increases in colanic acid production, and the growth of these strains was also affected (Figure 6C). The glucose consumption was also affected by the hydrolase. Glucose was completely consumed in the samples with hydrolase added at 0 h and 12 h, while the glucose remained at 1.82 g/L and 3.23 g/L in the samples with hydrolase added at 24 h and 36 h, respectively (Figure 6C). As expected, hydrolyzing the colanic acid capsule improved the growth of the strain and promoted the consumption of glucose by the strain, thereby increasing the production of colanic acid.

### 3.6. Scale-Up Production of Colanic Acid in a 3-L Bioreactor

**Figure 7:**
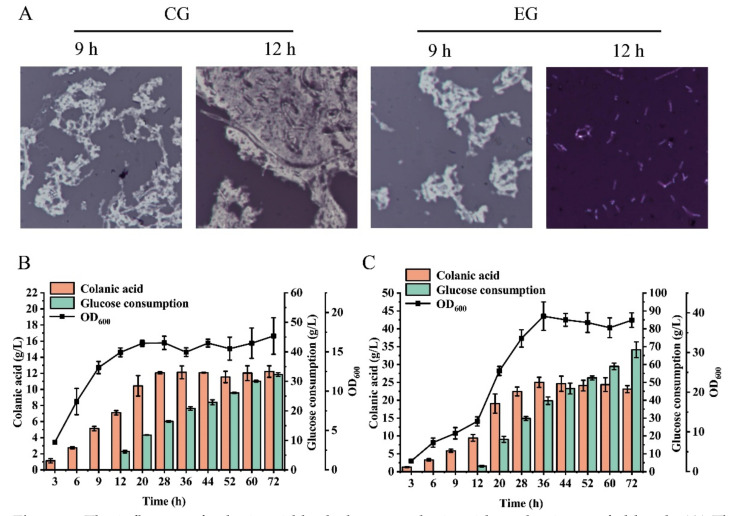
The influence of colanic acid hydrolase on colanic acid production on fed-batch. (**A**) The condition of the colanic acid capsule of SRP-4 strain on a 3-L bioreactor. A total of 4000 U/L colanic acid hydrolase was added to EG. CG, control group. EG, experimental group. (**B**) The colanic acid titer of SRP-4 on a 3-L bioreactor without adding the colanic acid hydrolase. (**C**) The colanic acid titer of SRP-4 on a 3-L bioreactor with 4000 U/L colanic acid hydrolase added. Both the CG and EG was supplemented glucose at 9 h and the glucose concentration was maintained between 9 and 12 g/L. The dissolved oxygen was maintained over 30%.

To verify large-scale industrial production, the strain was cultivated in a 3-L fed-batch bioreactor. The control group was cultured in the absence of colanic acid hydrolase, whereas the experimental group was cultured in the presence of 4000 U/L colanic acid hydrolase. Both the control and experimental group were supplemented with glucose at 9 h, and the glucose concentration was maintained at approximately 10 g/L. Similar to the control group, the colanic acid in the experimental group started to accumulate at 3 h and accumulated in large amounts at 9 h. By microscopy, we observed the colanic acid capsule layer at 9 h (Figure 7A). As the concentration of colanic acid increased, the solution became more viscous (Supplementary Video S2), and the dissolved oxygen level rapidly decreased from 80% to 30% at 9 h. Therefore, we added the colanic acid hydrolase to the 3-L bioreactor. The colanic acid capsule layer was completely hydrolyzed by 12 h (Figure 7A). However, in the control group, the colanic acid capsule layer was still observed (Figure 7A).

Due to the existence of the colanic acid capsule layer, the dissolved oxygen level in the fermentation broth of the control group was only 30%, while the dissolved oxygen level of the experimental group was 79% with the same rotation speed. The production of colanic acid in both the control and experimental group reached the highest at 36 h, which were 12.12 g/L and 24.99 g/L, respectively (Figure 7B,C). The colanic acid titer in the experimental group was 2.06-fold of the control group. The OD_600_ of the experimental group was the highest at 36 h, which was 39.15, whereas the OD_600_ of the control group was 16.14. The molecular weight of colanic acid was reduced due to the addition of hydrolase. After purification and measurement, the average molecular weight of colanic acid was 106.854 kDa, which was 1.2% of the control (8.71 MDa). Therefore, by adding colanic acid hydrolase, we increased the production of colanic acid to 24.99 g/L, which was 1249.5-fold of the initial titer (20 mg/L), and we obtained low-molecular-weight colanic acid.

## 4. Discussion

Colanic acid is usually produced by bacteria with unsatisfying conditions, such as low temperature, high osmotic pressure, and extreme pH [12,13,14]. Some studies have reported that when the lipopolysaccharide structure of the strain was incomplete, the RCS phosphorylation system was activated to promote the transcription of the colanic acid gene cluster [33,34]. At present, there is no strain with high colanic acid titer that was constructed by relieving the RCS phosphorylation system influence on colanic acid production. In our study, we achieved high production of colanic acid by relieving the regulation of RCS phosphorylation system influence on the synthesis of colanic acid, and increased the optimum fermentation temperature from 20 to 30 °C. We also found that the constructed strains can produce colanic acid at 37 °C. However, the colanic acid titer at 37 °C, which is the optimum temperature for strain growth, was significantly lower than that at 30 °C. Therefore, the production of colanic acid may be regulated by other mechanisms.

The synthesis of polysaccharides requires the participation of a variety of glycosyl donors, so enhancing the precursors or knocking out the competing pathways of polysaccharide synthesis can increase the titer of polysaccharides. Such as hyaluronic acid [32]. We enhanced the precursor supply for colanic acid synthesis, but the titer increase was low compared with the SR series strains. We speculate that, because the key genes related to the synthesis of colanic acid are located in the colanic acid gene cluster, enhancing the supply of precursors alone cannot significantly increase the production of colanic acid. Therefore, the effects of the precursors can only be manifested when the regulation of colanic acid synthesis is relieved by the RCS phosphorylation system.

In the RCS phosphorylation system, phosphorylated RcsB is necessary for transcription of the colanic acid gene cluster [17]. However, in the absence of external stimulus, phosphorylated RcsB is dephosphorylated by RcsC and RcsD, which maintains a low level of phosphorylated RcsB expression [39,40]. RcsA is an auxiliary activator protein that acts with RcsB [14]. Therefore, by increasing the expression of RcsA protein, RcsB can easily bind to RcsA protein and active the transcription of *wza* gene cluster [41].

The high concentration of high-molecular-weight colanic acid in the solution forms the colanic acid capsule layer, and inhibits the growth of the strain and the acquisition of external nutrients by the strain. During fermentation, we also found that high concentrations of colanic acid not only inhibited the growth of the strain, but also reduced the dissolved oxygen level of the fermentation broth. The synthesis of glycosidic bonds during polysaccharide synthesis requires ATP for energy, and ATP is only produced in large quantities when the oxygen level is sufficient. Unfortunately, this phenomenon has not been paid attention. After adding hydrolase, the dissolved oxygen level in the fermentation broth increased. Therefore, after adding hydrolase, high colanic acid production may have been the result of the combined effects of improved strain growth, glucose acquisition, and dissolved oxygen. Our results demonstrate that the addition of colanic acid hydrolase can effectively increase the production of colanic acid. This strategy will be helpful in the fermentative production of other polysaccharides.

## 5. Conclusions

In this work, we successfully constructed a strain capable of producing low molecular weight colanic acid at 30 °C by relieving the regulation of RCS system on colanic acid synthesis, increasing copies of genes involved in precursor synthesis, expressing and adding hydrolase, and media optimization. After adding 4000 U/L colanic acid hydrolase, the colanic acid titer on 3-L bioreactor reached 24.99 g/L, which was 1249.5-fold of the wild-type K-12 in shake flasks, and was the highest colanic acid titer reported so far. The molecular weight of hydrolyzed colanic acid was 106.854 kDa, which was 98.7% lower than the initial molecular weight. In addition, we also found that the capsular layer formed by high-molecular-weight colanic acid polysaccharide would seriously inhibit the growth of the strain and the increase in colanic acid titer, and this phenomenon was relieved by adding hydrolase. Overall, our results proved that we achieved the goal of high production of low molecular weight colanic acid, and we believe this will lay the foundation for large-scale production of colanic acid. Here the developed strategies may also be used for the production of the other polysaccharides.

## Figures and Tables

**Table 1:** Log2 ratio of *wza* gene expression in SR-series strains to that in the *E. coli* K-12.

Strains	Ratio
SR-1	2.16
SR-2	1.32
SR-3	1.14
SR-4	2.38
SR-5	1.39
SR-6	2.41
SR-7	3.61

**Table 2:** The molecular weight of colanic acid produced at different temperature by strain SRP-4.

Temperature (°C)	The Average Molecular Weight of Colanic Acid (MDa)
20	9.87
25	9.85
30	8.71
37	9.33

## Data Availability

All data are available in the manuscript.

## Supplementary Materials

The following supporting information can be downloaded at: https://www.mdpi.com/article/10.3390/microorganisms10050877/s1, Figure S1: Media optimization; Figure S2: Purification of the colanic acid hydrolase; Figure S3: Colanic acid capsule layer inhibited strain growth and glucose acquisition; Figure S4: The effect of adding different amounts of colanic acid hydrolase on colanic acid production; Table S1: Bacterial strains and plasmids used in this study; Table S2: Primers used in this study; Video S1: The fermentation broth of SRP-4; Video S2: The state of the fermentation broth of strain SRP-4 in the 3-L bioreactor.
