# Supplementary material for: Combinatorial Metabolic Engineering and Enzymatic Catalysis Enable Efficient Production of Colanic Acid
Source: Microorganisms. 2022 Apr 22;10(5):877. doi: 10.3390/microorganisms10050877 (PMC9143390; doi:10.3390/microorganisms10050877)
Supplement: Supplementary file 1 [file microorganisms-10-00877-s001.zip › microorganisms-1631826 supp proof.pdf]

Table S1: Bacterial strains and plasmids used in this study

Strain or plasmid	Description	Sources
strains		
JM109	Wild-type *E. coli*	ATCC
BL21(DE3)	Wild-type *E. coli*	ATCC
MG1655	Wild-type *E. coli* K12; F- λ- rph-1	ATCC
SC-1	*E. coli* K12 △*wcaj*	This work
SR-1	*E. coli* K12 △*lon*	This work
SR-2	*E. coli* K12 △*hns*	This work
SR-3	*E. coli* K12 *mota*::*rcsa*	This work
SR-4	*E. coli* K12 △*lon* △*hns*	This work
SR-5	*E. coli* K12 △*lon* *mota*::*rcsa*	This work
SR-6	*E. coli* K12 △*hns* *mota*::*rcsa*	This work
SR-7	*E. coli* K12 △*lon* △*hns* *mota*::*rcsa*	This work
SP-1	*E. coli* K12 *flie*::*galu*	This work
SP-2	*E. coli* K12 *flie*::*galu* *flgg*::*gale*	This work
SP-3	*E. coli* K12 *flie*::*galu* *flgg*::*gale* *flga*::*gmd*	This work
SP-4	*E. coli* K12 *flie*::*galu* *flgg*::*gale* *flga*::*gmd* *flit*::*manc*	This work
SP-5	*E. coli* K12 *flie*::*galu* *flgg*::*gale* *flga*::*gmd* *flit*::*manc* *flir*::*manb*	This work
SRP-1	*E. coli* K12 *flie*::*galu* *flgg*::*gale* *flga*::*gmd* *flit*::*manc* *flir*::*manb* *mota*::*rcsa*	This work
SRP-2	*E. coli* K12 *flie*::*galu* *flgg*::*gale* *flga*::*gmd* *flit*::*manc* *flir*::*manb* *mota*::*rcsa* △*lon*	This work
SRP-3	*E. coli* K12 *flie*::*galu* *flgg*::*gale* *flga*::*gmd* *flit*::*manc* *flir*::*manb* *mota*::*rcsa* △*hns*	This work
SRP-4	*E. coli* K12 *flie*::*galu* *flgg*::*gale* *flga*::*gmd* *flit*::*manc* *flir*::*manb* *mota*::*rcsa* △*lon* △*hns*	This work
BL21-CAE	BL21(DE3) bearing PACY-CAE	This work
Plasmid		
pCas9	*repA101*(Ts) *kan* Pcas-*cas9* ParaB- *Red* *lacIq* Ptrc-*sgRNA-Pmb1*	This work
pTarget	pMB1 aadA sgRNA	This work
PACYDUET1	p15A CmR PT7 T7 terminator PlacI-lacI	This work
PCAY-CAE	p15A CmR PT7-*cae* PlacI-*lacI*	This work
pTarget-*flie*	pMB1 aadA sgRNA-*flie*	This work
pTarget-*flgg*	pMB2 aadA sgRNA-*flgg*	This work
pTarget-*flga*	pMB3 aadA sgRNA-*flga*	This work
pTarget-*flit*	pMB4 aadA sgRNA-*flit*	This work
pTarget-*flir*	pMB5 aadA sgRNA-*flir*	This work
pTarget-*mota*	pMB6 aadA sgRNA-*mota*	This work
pTarget-*lon*	pMB7 aadA sgRNA-*lon*	This work
pTarget-*hns*	pMB8 aadA sgRNA-*hns*	This work

Table S2: Primers used in this study

Names	Sequence (5'-3')
PACY-T	TTAACCTAGGCTGCTGCCACC
PACY-B	ATATCTCCTTATTATTTCTACAGGGGAATTGTTATCCGCT
CAE-T	TAGAAATAATAAGGAGATATACCATGGGCCATCAC

CAE-B	GTGGCAGCAGCCTAGGTTAATTAGATGTAAGCCACCGGGTAGGTA
pTarget-*wcaj*-L	GTCAGCACATTGATAAACTGGTTTTAGAGCTAGAAATAGCAAGTT
pTarget-*wcaj*-R	CAGTTTATCAATGTGCTGACACTAGTATTATACCTAGGACTGAGC
T-*wcaj*-L	TGCAGACCGGGGCGATAAATTTT
T-*wcaj*-R	GGGCTAATAACAGGAACAACGTATGAGCTTACGTGAAAAAACCATCAGCG
D-*wcaj*-L	TTTCACGTAAGCTCATACGTTGTTCCTGTTATTAGCCCCTTAC
D-*wcaj*-R	GAAAAAAATCCCGGCGCGAAGA
pTarget-*lon*-L	TAGAACCGATGTAAGTACGGGTTTTAGAGCTAGAAATAGCAAGTT
pTarget-*lon*-R	CCGTACTTACATCGGTTCTAACTAGTATTATACCTAGGACTGAGC
T-*lon*-L	CGCAGGTTGAACCGGAAGATC
T-*lon*-R	TTGCGCGAGGTCAAGAGCTCTCTCTTAGTTTAATTTCCGCC
D-*lon*-L	GAAATTAAACTAAGAGAGAGCTCTTGACCTCGCGCAAAATGCAC
D-*lon*-R	GACAAGAAACGGGGGCAATTGT
pTarget-*hns*-L	CGCGAAATGCTGATCGCTGAGTTTTAGAGCTAGAAATAGCAAGTT
pTarget-*hns*-R	TCAGCGATCAGCATTTCGCGACTAGTATTATACCTAGGACTGAGC
T-*hns*-L	GATTGCAAAGGCGTTGAATTAGC
T-*hns*-R	CAAACCACCCCAATATAAGTTTGAGGATTGCACTTGCTTAAAATCCCG
D-*hns*-L	CGGGATTTTAAGCAAGTGCAATCCTCAAACTTATATTGGGGTGGTTTG
D-*hns*-R	GTATATGCGTTCTCCCTTACGAAG
pTarget-*mota*-L	GCCGCAACAATACCAAACGCGTTTTAGAGCTAGAAATAGCAAGTT
pTarget-*mota*-R	GCGTTTGGTATTGTTGCGGCACTAGTATTATACCTAGGACTGAGC
T-*mota*-L	CGATGTTCGGCTGCTTATTCACTTC
T-*mota*-R	TCCTTGTGTGAGTTAATCTTAAGGGTCTGATCACATTATACGAGCCGATGATTAATTGTCAAACTTTCCTCGGCATTTTATTGGCTTACG
M-*rcsa*-L	GCTCGTATAATGTGATCAGACCCTTAAGATTAACTCACACAAGGAGATATACCATGTCAACGATTATTATGGATTTATGTAGTTACACCC
M-*rcsa*-R	GTTGATGGCGAAATCTTAGCGCATGTTGACAAAAATACCATTAGTCA
D-*mota*-L	ATTTTTGTCAACATGCGCTAAGATTTCGCCATCAACCGATAAAGCAG
D-*mota*-R	ACTTTCCAGCTGCAACTGCTG
pTarget-*flie*-L	AGCGATACAGGGGATTGAAGGTTTTAGAGCTAGAAATAGCAAGTT
pTarget-*flie*-R	CTTCAATCCCCTGTATCGCTACTAGTATTATACCTAGGACTGAGC
T-*flie*-L	ATTAAATTATCCAGAGGTTACTGTTGCGTCT
T-*flie*-R	AAGAAGAGATGGGCATTAAGAAGTAAGGCTACGGCGATGAGTGC
M-galu-L	GCCGTAGCCTTACTTCTTAATGCCCATCTCTTCTTCAAGC
M-*galu*-R	ATCGGCTCGTATAATGTGATCAGACCTTTGTTTAACTTTAAGAAGGAGATATACCATGGCTGCCATTAATACGAAAGTCAAAAAA

D-*flie*-L	TCCTTCTTAAAGTTAAACAAAGGTCTGATCACATTATACGAGCCGATGATTAATTGTCAATCTCGTCTCCCGGATAATTTCTGG
D-*flie*-R	GCAGACGCAGTTCGTGAACTTT
pTarget-*flgg*-L	GATGTCGCGATTAAAGGGCAGTTTTAGAGCTAGAAATAGCAAGTT
pTarget-*flgg*-R	TGCCCTTTAATCGCGACATCACTAGTATTATACCTAGGACTGAGC
T-*flgg*-L	AAATCACTATTGCTGCCGATGGC
T-*flgg*-R	TCCTTCTTAAAGTTAAACAAAGGTCTGATCACATTATACGAGCCGATGATTAATTGTCAAAAGTGCATATGTCTTCTTGCCGG
M-gale-L	ATCGGCTCGTATAATGTGATCAGACCTTTGTTTAACTTTAAGAAGGAGATATACCATGAGAGTTCTGGTTACCGGTGG
M-*gale*-R	CGCCACGTTGATTAATCGGGATATCCCTGTGGATGGC
D-*flgg*-L	CACAGGGATATCCCGATTAATCAACGTGGCGGAAGAACTG
D-*flgg*-R	TAGCGCGGCACAGTATCAAAG
pTarget-*flga*-L	TTGATATCCAGCACCGTACGGTTTTAGAGCTAGAAATAGCAAGTT
pTarget-*flga*-R	CGTACGGTGCTGGATATCAAACTAGTATTATACCTAGGACTGAGC
T-*flga*-L	GACGTGTCATACGATCAGTTACTCTG
T-*flga*-R	CTGGAGTCATAAGAAGGTCAGGCGCTGAACAATG
M-gmd-L	GTTCAGCGCCTGACCTTCTTATGACTCCAGCGCGATCG
M-*gmd*-R	ATCGGCTCGTATAATGTGATCAGACCTTTGTTTAACTTTAAGAAGGAGATATACCATGTCAAAAGTCGCTCTCATCACC
D-*flga*-L	TCCTTCTTAAAGTTAAACAAAGGTCTGATCACATTATACGAGCCGATGATTAATTGTCAAAAAAAAGTTGTGCAATTGCGATGTG
D-*flga*-R	AATACGGTATTGCAGTTCTGCGG
pTarget-*flit*-L	CGAAATGGCGTATGTGAATGGTTTTAGAGCTAGAAATAGCAAGTT
pTarget-*flit*-R	CATTCACATACGCCATTTCGACTAGTATTATACCTAGGACTGAGC
T-*flit*-L	AGCAGTTCGAAAACAACAGTAATTCCAAG
T-*flit*-R	TCCTTCTTAAAGTTAAACAAAGGTCTGATCACATTATACGAGCCGATGATTAATTGTCAAGCCAGGCGAAATATAAATGCGG
M-manc-L	ATCGGCTCGTATAATGTGATCAGACCTTTGTTTAACTTTAAGAAGGAGATATACCATGGCGCAGTCGAAACTCTATC
M-*manc*-R	GCCATAGGCACTTAATTACACCCGTCCGTAGCGATC
D-*flit*-L	CTACGGACGGGTGTAATTAAGTGCCTATGGCGATCAGGG
D-*flit*-R	ATTTACACGCTGCACGCGAATA
pTarget-*flir*-L	GCGTTCGCTCAGAATCGGCGGTTTTAGAGCTAGAAATAGCAAGTT
pTarget-*flir*-R	CGCCGATTCTGAGCGAACGCACTAGTATTATACCTAGGACTGAGC
T-*flir*-L	GATTATCGACCTGGTGATAGCCAG
T-*flir*-R	TCCTTCTTAAAGTTAAACAAAGGTCTGATCACATTATACGAGCCGATGATTAATTGTCAACGGCCAGAAGTACAGGTTTAACC
M-manb-L	ATCGGCTCGTATAATGTGATCAGACCTTTGTTTAACTTTAAGAAGGAGATATACCATGAAAAAATTAACCTGCTTTAAAGCCTATGAT

M-*manb*-R	ATACGATTAAGTAAACCTAATGCCATTACTCGTTCAGCAACGTCAGC
D-*flir*-L	CGTTGCTGAACGAGTAATGGCATTAGGTTTACTTAATCGTATGGCC
D-*flir*-R	TCCATAATAATCGTTGACATGGCATACCC
reca-T-qPCR	CATCCATGGAACGGTCTTCACC
reca-B-qPCR	ATGGCTATCGACGAAAACAAACAGAAA
wza-T-qPCR	ATGATGAAATCCAAAATGAAATTGATGCCAT
wza-B-qPCR	TTGATGACGTCTTTGCCCATCG

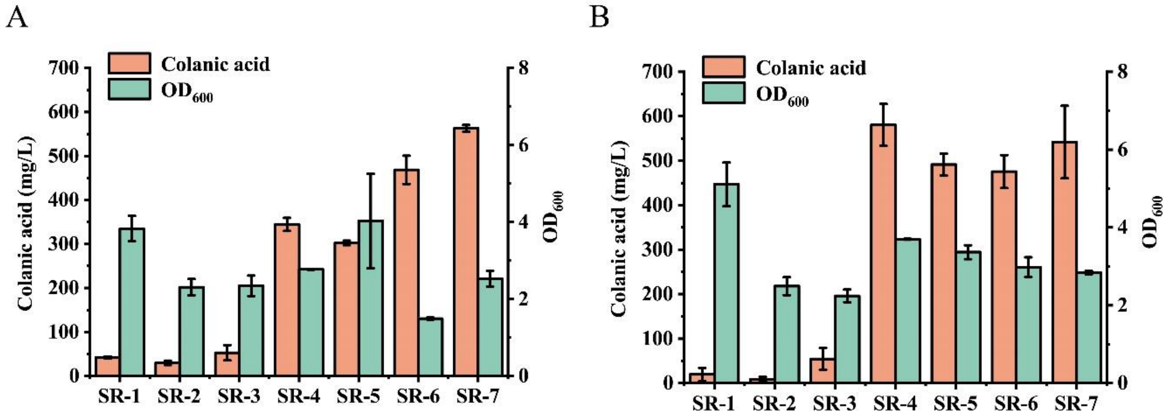

Supplementary Figure S1 Media optimization. (A) Colanic acid titers of SR recombinant strains in LB medium at 30°C in shake flasks. **(B)** Colanic acid titers of SR recombinant strains in SOB medium at 30°C in shake flasks.

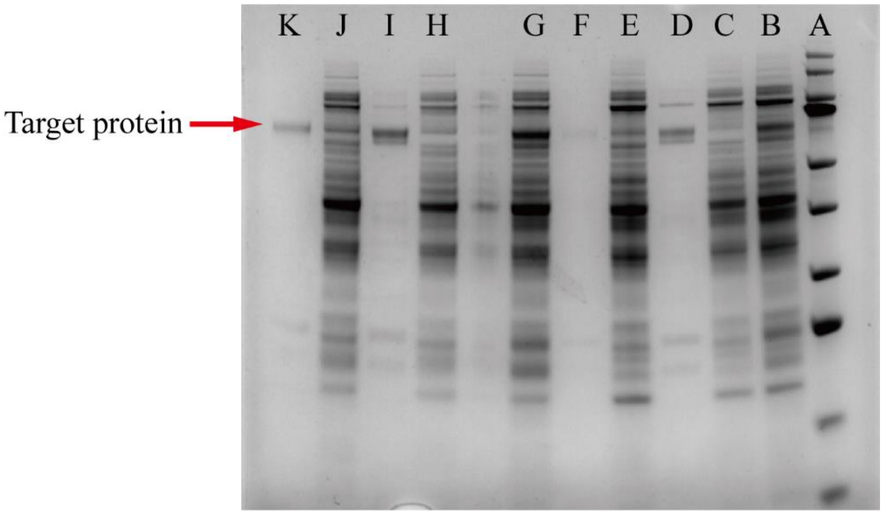

Supplementary Figure S2 Purification of the colanic acid hydrolase. A was the marker. B, C, D, E and F were the control samples without expression of colanic acid hydrolase. After loading onto an equilibrated His GraviTrap column (GE Healthcare, US) and washing with buffer A (500 mM NaCl, 20 mM PB, 20 mM imidazole, pH 8.0), sample B was eluted with 20% B buffer (500 mM NaCl, 20 mM PB, 500 mM imidazole, pH 8.0.). After loading onto an equilibrated His GraviTrap column and washed with buffer A, sample C was eluted with 4% buffer B. After loading onto an equilibrated His GraviTrap column and washing with 4% buffer B, sample D was eluted with 20% buffer B. After loading onto an equilibrated His GraviTrap column and washing with buffer A, sample E was eluted with 10% buffer B. After loading onto an equilibrated His GraviTrap column and washing with 10% buffer B, sample F was eluted with 20% B buffer. G, H, I, J and K were experimental samples with expression of colanic acid hydrolase. The purified process of G, H, I, J and K were the same as B, C, D, E and F, respectively.

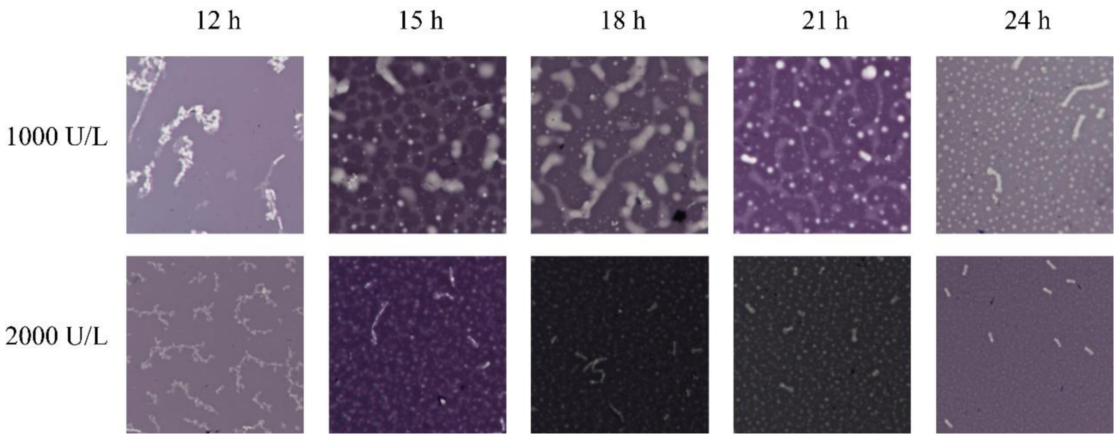

Supplementary Figure S3 Colanic acid capsule layer inhibited strain growth and glucose acquisition. After adding colanic acid hydrolase, the capsule was disrupted. The cells were released from the colanic acid capsule. The large white and spherical ones were colanic acid.

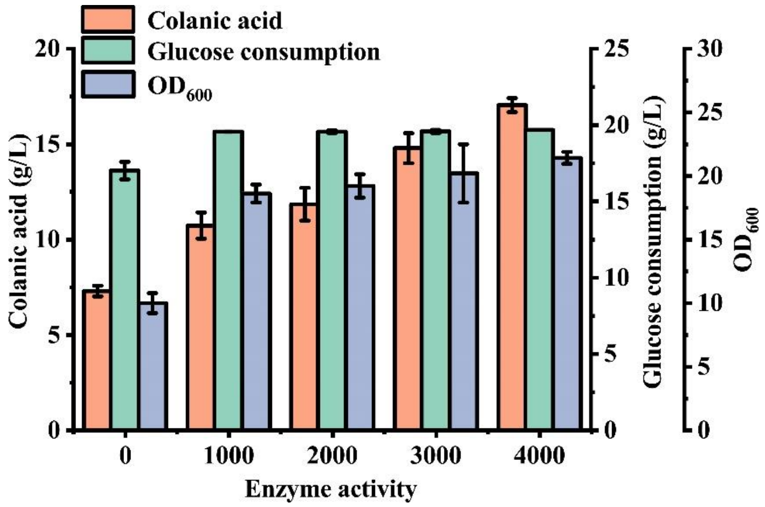

Supplementary Figure S4 The effect of adding different amounts of colanic acid hydrolase on colanic acid production. SRP-4 strain was cultivated in TBG medium at 30°C, 220 rpm in shake flasks for 48 h. 1000 U/L, 2000 U/L, 3000 U/L and 4000 U/L colanic acid hydrolase were added at 12 h.
